# Factors Associated with Tuberculosis Mortality in Manjung District, Perak, Malaysia

**DOI:** 10.21315/mjms2023.30.3.15

**Published:** 2023-06-27

**Authors:** Asraf Ahmad Qamruddin, Gregory Xavier, Syed Mohammad Zahid

**Affiliations:** Manjung District Health Office, Perak, Malaysia

**Keywords:** tuberculosis, tuberculosis mortality, risk factors

## Abstract

**Background:**

Tuberculosis (TB) is a communicable disease which contributes to a major cause of ill health. Worldwide, it is one of the leading causes of death from a single infectious agent.

**Objectives:**

The study aimed to describe the epidemiology and factors associated with TB mortality in Manjung district, Perak, Malaysia.

**Methods:**

All confirmed TB cases from 2015 to 2020 registered in Manjung district under Sistem Maklumat Tibi (MyTB) were included. Factors associated with TB mortality were analysed by using simple and multiple logistic regression analysis.

**Results:**

A total of 742 TB cases were included in the analysis, from which 121 cases (16.3%) died before completing their treatment. The highest death was reported in 2020 (25.7%) and the lowest in 2019 (12.9%). From multiple logistic regression analysis, age 45 years old–64 years old (adjusted OR = 3.62; 95% CI: 1.38, 9.54), > 65 years old (adjusted OR = 8.67; 95% CI: 3.17, 23.74), non-Malaysian (adjusted OR = 5.18; 95% CI: 2.04, 13.14), cases notified by government hospitals (adjusted OR = 6.78; 95% CI: 3.04, 15.09), HIV-positive status (adjusted OR = 8.60; 95% CI: 3.58, 20.67) and HIV testing not offered/unknown (adjusted OR = 2.58; 95% CI: 1.18, 5.62) were significantly associated with TB mortality.

**Conclusion:**

This study found that TB patients who were 45 years old and above, positive HIV, late diagnosis and are foreigners had a higher risk for TB mortality. Early diagnosis, optimised screening and close monitoring should be practised to reduce TB mortality.

## Introduction

Tuberculosis (TB) is a communicable disease that contributes to a major cause of ill health and is one of the leading causes of death worldwide from a single infectious agent (1). It is caused by *Mycobacterium tuberculosis*, which typically affects the lungs (pulmonary TB). It can also affect other sites; however, it is curable and preventable (2).

The ‘end TB strategy’ milestone for TB death in 2020 by the World Health Organization (WHO) was a reduction by 35% of the total TB death as compared to the 2015 baseline. However, only a 9.2% reduction was achieved, which was about one-quarter of the way to the milestone. The COVID-19 pandemic caused disruptions to the diagnosis and treatment of all diseases, including TB (3). However, it only partially explained that the compromise on the milestone progress achievement up to 2019 was only 14% as compared to the 2015 baseline, which was way below the 35% target (1).

In Malaysia, the estimated TB incidence rate in 2019 was 92 cases per 100,000 population, with an estimated TB mortality rate of 4 cases per 100,000 population (4). The TB mortality rate in Malaysia was 5.5 per 100,000 population in 2015 (5). Studies examining factors associated with TB were carried out worldwide in both high and low TB incidence countries. Many well-recognised factors that contributed to TB mortality were found. In Malaysia, studies that investigated TB death and its associated factors are still limited.

Manjung is a district in the south-western part of Perak, Malaysia, with a total population of 245,683. The most common ethnic groups are Malays (57.5%), Chinese (28.4%), Indians (12.6%) and others (1.5%) (6). The major sectors of economy in Manjung district are agriculture and tourism. Manjung is the third biggest district in Perak, with one of the highest TB cases in Perak. To the best of knowledge, there is no published study on TB death situation in Manjung district. Therefore, this study was conducted to describe the epidemiology of TB mortality and its associated factors with TB patients on treatment in Manjung district from 2015 to 2020.

## Methods

A case-control study was conducted between 1 May and 9 June 2022 for all confirmed TB cases reported to the Manjung district Health Office. Cases were registered in the national online system called Sistem Maklumat Tibi (MyTB) over 6 years (2015–2020).

In Malaysia, all confirmed TB cases in government or private healthcare facilities are required by law to be reported under the Prevention and Control of Infectious Diseases Act 1988 (Act 342) (7). MyTB was developed and is run by the Ministry of Health, Malaysia to standardise the reporting, investigation, and findings at the district, state, and national levels for the control and prevention of TB.

**Figure 1:**
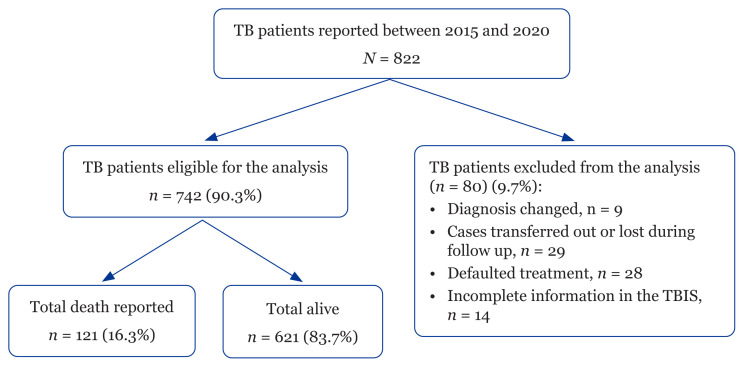
Flow diagram of 822 patients with TB reported to the Malaysia TBIS between 2005 and 2010

From the extracted TB cases, excluded cases from the analysis were patients whose diagnosis changed, cases transferred out or lost during follow-up, defaulted treatment and incomplete information in the Tuberculosis Information System (TBIS) (Figure 1).

### Definitions

Cases were defined as all-cause TB mortality amongst TB patients before they completed their anti-TB treatment. Controls were TB patients who were alive until they completed their anti-TB treatment or until the study ended. All-cause TB mortality was included as per the definition of TB mortality from the *Malaysian Clinical Practice Guidelines on Tuberculosis* (8).

The notification centre was categorised into three groups based on the institutions that had diagnosed and notified the TB cases, such as: i) government health clinics; ii) private health clinics and iii) hospitals.

Active case detection was when contacts of newly diagnosed patients were systematically screened. Patients presenting to health services with symptoms and later being confirmed of TB were termed as passive case detection. Meanwhile, TB cases detected from various screening, such as workers screening, medical screening and screening of TB in HIV and diabetes patients, were termed as detection from screening (9).

Extrapulmonary TB is defined as TB diagnosed in organs other than the lungs, such as the lymph nodes, pleura, gastrointestinal tract or central nervous system (10). Smear-positive pulmonary TB (PTB) is defined as a PTB patient with at least one or more initial sputum smear examinations (direct smear microscopy) positive for acid-fast bacilli (AFB) or with one sputum specimen positive for AFB and radiographic abnormalities consistent with active PTB, or with one sputum specimen positive for AFB and culture positive for *Mycobacterium tuberculosis* (11). Smear-negative PTB is defined as a PTB patient with at least three negative results in direct smear sputum microscopy but with radiographic results suggestive of active TB or sputum culture positive for *Mycobacterium tuberculosis* (11).

### Statistical Analysis

Data collection and analysis were conducted at the Manjung district Health Office. Data were downloaded from MyTB. This was followed by importing and analysing the data by using IBM Statistical Package for the Social Sciences (SPSS) version 24.0 software.

Non-normally distributed continuous variables were presented as median (interquartile range), while categorical variables were presented as numbers (percentage). The risk factors associated with TB mortality diagnosis were evaluated through univariate and multivariate logistic regression. Variables with a *P*-value of less than 0.05 from the univariate analysis were selected and considered for multiple logistic regression analysis. Multicollinearity and interaction were checked for the final model. Fitness of the model was tested by using the Hosmer-Lemeshow goodness-of-fit test, classification table and area under the receiver operating characteristics (ROC) curve in SPSS software. All TB cases from 2015 to 2020 were analysed.

The significance level for all statistical tests was set at 0.05 unless otherwise stated.

## Results

### Characteristics of the Reported Cases

**Figure 2:**
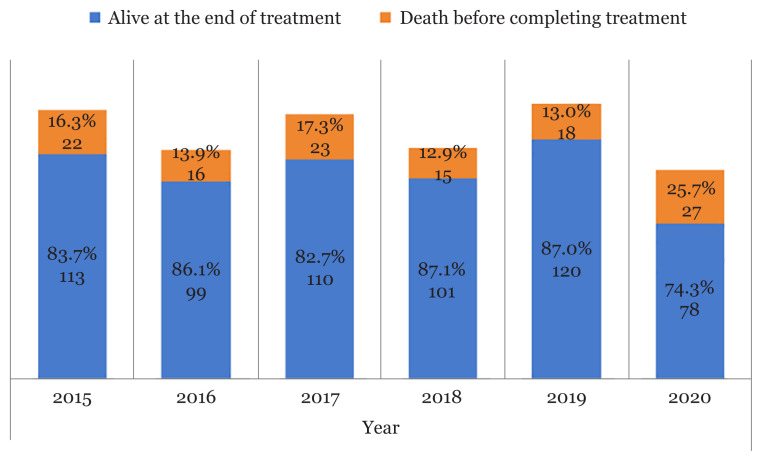
Cases with patients who were alive and died at the end of treatment according to year

Of the 822 patients with the diagnosis of TB reported in the Manjung district in the TBIS from 2015 to 2020, 742 (90.3%) met the eligibility criteria for the study and 80 cases were discarded, as shown in Figure 1. About 121 (16%) of patients died before completing their TB treatment, but 34 were audited to have died due to TB. A total of 621 (83.7%) were still alive. The case-control analysis included those who died with or due to TB and as compared with those who remained alive during the study. Figure 2 shows the number of patients who were alive at the end of treatment and patients who died before completing treatment according to year. The highest TB mortality percentage was in 2020 (25.7%) and the lowest was in 2018 (12.9%). Table 1 shows characteristics of the 742 reported TB cases in Manjung district as well as simple and multiple logistic regression analyses of factors associated with TB death in Manjung district during the study.

### Factors Associated with TB Mortality

From the multiple logistic regression analysis, age 45 years old–64 years old (*P* = 0.002) and above 65 years old (*P* < 0.001), non-Malaysian (*P* = 0.002), cases notified by government hospital (*P* < 0.001), HIV status at the time of diagnosis positive (*P* < 0.001) or testing not offered/unknown (*P* = 0.004) were significantly associated with TB mortality after adjusting for other factors. The Hosmer-Lemeshow goodness-of-fit test showed that the model was fit with a *P* -value of 0.468. Overall percentage of the classification table was 84% and area under the ROC curve was 0.845 (95% CI: 0.784, 0.908).

## Discussion

It was found that between 2015 and 2020, 16.3% of TB patients living in Manjung district died before completing their treatments. This was higher than the Malaysian reported TB mortality of 10.2% between 2014 and 2017 (12). The highest TB mortality was in 2020 when 25.7% of cases died. This was the year when the COVID-19 pandemic started and impact of the pandemic had reversed years of global progress in reducing the number of people who died from TB. According to the WHO, the number of estimated TB deaths in 2020 worldwide was back to the level of before 2017 (13).

In this study group, four factors were significantly associated with TB mortality based on statistical adjustments from other significant factors. The four factors were: i) age; ii) citizenship status; iii) notification centre and iv) HIV status at the time of diagnosis.

As compared to 15 years old–24 years old age group, the 45 years old–64 years old age group and above 65 years old age group had 3.62 times and 8.67 times higher odds respective of TB mortality. A study of TB mortality in the United States between 2009 and 2013 supported this finding with the 45 years old–64 years old age group, with 2.57 times higher odds and above 65 years old with 5.76 higher odds (14). A retrospective cohort study also concluded that the hazard ratio increased by 1:1 with each additional year for TB mortality (15). Older people were more likely to present with atypical findings. They also often had one or more comorbidities, such as diabetes mellitus, chronic obstructive pulmonary disease or lung cancer which could increase the risk of TB and mortality (16). In this study, both TB mortality that died due to TB or other causes during TB treatment were included. Older people were more likely to die from other causes while being treated for TB, which contributed to the higher observed odds.

Non-Malaysian were more than five times more likely to die during TB treatment as compared to Malaysian citizens. Studies showed that immigrants had higher TB mortality for infectious diseases as compared to locals (17, 18). Non-citizens were more vulnerable to death from infectious diseases, such as TB, due to a series of risk factors associated with migration. The migrants in Manjung district are majority labour class workers; hence, their exposure risk in their living conditions predisposed them to be infected with TB. They are more likely to be diagnosed in the later stage of the infection; therefore, increasing the risk of TB mortality. Problems and barriers in accessing healthcare and lack of knowledge about the healthcare system might also contribute to higher TB mortality amongst them (19).

Interestingly, notification centre was significantly associated with TB mortality. As compared to TB cases notified by government clinics, cases notified by government hospitals were 6.78 times more likely to die during treatment. TB cases that were presented to government hospitals tended to be more severe cases that required hospitalisation. Whereas, government clinics primarily detected TB cases from the screening of symptomatic or close contact with TB cases, which were clinically more stable. Studies showed that the TB mortality rate was about 52% higher amongst those late presenters than in early presenters (20).

Several previous studies reported similar results to those found in the present study regarding HIV and TB mortality. HIV-positive statuses were more than nine times to die during TB treatment. Meanwhile, those with testing not offered or unknown were almost three times more likely to die during TB treatment. A study conducted by Marks et al. (21) with the National Tuberculosis Surveillance System data from 1997 to 2005 found that patients infected with HIV had higher odds of TB diagnosis at death and death during TB treatment than patients who were HIV-negative. Later, a similar study conducted by Hannah et al. (14) by using data from 2009–2013 reported similar findings. A meta-analysis reported that HIV-positive had a hazard ratio of 2:6 for TB mortality (22). HIV increased the risk of TB infection and severity of TB disease and mortality. Furthermore, TB may also act as a cofactor in the progression of HIV infection by increasing the HIV viral load (23).

Since the study population consisted of the population in the Manjung district, the result cannot be generalised to other populations. Due to the limitation of secondary data, the study had no access to external information other than the online notification database. For a better understanding of associated factors with TB mortality, future researchers may employ other study designs, such as cohort studies.

Based on this study, it is recommended that screening and awareness for TB in primary health care should be optimised, especially amongst the high-risk groups. These groups comprise the elderly, those with HIV/high-risk behaviours and foreigners. The lack of screening and awareness in primary healthcare would contribute to late presentation to hospitals, increasing the risk of TB mortality. Adjunct to primary healthcare screening, contact tracing and follow-up of close contacts should also be enhanced. Employers of foreign labour class workers should take initiatives to provide better healthcare accessibility to ensure they are not marginalised from mainstream healthcare services.

## Conclusion

About 1.6 in 10 TB patients died during the treatment course in Manjung district. Older age, non-Malaysian, cases notified by the hospital and positive HIV status were significant factors associated with TB mortality. To strengthen and reduce the TB mortality rate in Manjung district, targeted approaches, such as early diagnosis, optimised screening and close monitoring, should be practised to reduce TB mortality within this group.

## Figures and Tables

**Table 1:** Sociodemographic, simple and multiple logistic regression analysis of factors associated with TB death in Manjung district from 1 January 2015 to 31 December 2020 (*N* = 742)

Variable	*n* (%)	Wald statistic	df^a^	Crude OR^b^ (95% CI^c^)	*P*-value	Adj. OR^b^ (95% CI^c^)	*P*-value
Sociodemographics							
Age (years old) d							
< 14	13 (1.8%)			0.01 (0.01, 0.01)	0.999	0.01 (0.01, 0.01)	0.999
15–24	89 (12.0%)			1		1	
25–44	254 (34.2%)	21.843	4	2.14 (0.87, 5.28)	0.099	1.98(0.74, 5.30)	0.175
45–64	270 (36.4%)			2.84 (1.17, 6.90)	0.002	3.62 (1.38, 9.54)	0.009
> 65	116 (15.6%)			5.98 (2.39, 14.98)	< 0.001	8.67 (3.17, 23.74)	< 0.001
Gender							
Female	284 (38.3%)	7.267	1	1			
Male	458 (61.7%)			1.81 (1.18, 2.78)	0.007		
Citizenship d							
Malaysian	713 (96.1%)	9.297	1	1		1	
Non-Malaysian	29 (3.9%)			3.35 (1.54, 7.29)	0.002	5.18 (2.04, 13.14)	0.001
Education level							
None/Primary	65 (8.8%)	7.153	2	1			
Secondary	607 (81.8%)			0.60 (0.33, 1.11)	0.102		
Tertiary	70 (9.4%)			0.24 (0.08, 0.68)	0.008		
Case detection							
Passive	52 (7.0%)	1.021	2	1			
Active	667 (89.9%)			0.91 (0.42, 1.99)	0.814		
Screening	23 (3.1%)			0.48 (0.11, 2.06)	0.322		
Notification centre d							
Government clinic	187 (25.2%)	33.423	2	1		1	
Private facility	492 (66.3%)			0.42 (0.05, 3.44)	0.415	0.42 (0.05, 3.51)	0.422
Government hospital	63 (8.5%)			7.67 (3.50, 16.79)	< 0.001	6.78 (3.04, 15.09)	< 0.001
Diabetes							
No	522 (70.4%)	190.755	1	1			
Yes	220 (29.6%)			1.01 (0.66, 1.54)	0.978		
HIV status at time of diagnosis d							
Negative	677 (91.2%)	270.812	2	1		1	
Positive	27 (3.6%)			9.13 (4.11, 20.29)	< 0.001	8.60 (3.58, 20.67)	< 0.001
Testing not offered/unknown	38 (5.1%)			2.90 (1.41, 5.94)	0.004	2.58 (1.18, 5.62)	0.017
Smoking status							
No	503 (67.8%)	190.830	1	1			
Yes	239 (32.2%)			1.25 (0.831, 1.876)	0.286		
BCG scar							
No	91 (12.3%)	23.416	1	1			
Yes	651 (87.7%)			0.61 (0.36, 1.03)	0.064		
TB category							
Smear-negative PTB	161 (21.7%)	48.207	2	1			
Smear-positive PTB	508 (68.5%)			0.71 (0.46, 1.12)	0.144		
Extra PTB	73 (9.8%)			0.55 (0.25, 1.21)	0.135		
Healthcare workers							
No	722 (97.3%)	261.730	1	1			
Yes	20 (2.7%)			0.56 (0.13, 2.46)	0.445		

Notes:

a Constant −4.576;

b Forward LR and Enter-Manual method applied. Final output from Enter-Manual method (variables with *P*-value less than 0.05 from univariate analysis were manually entered);

c No multicollinearity and no interaction detected;

d Hosmer-Lemeshow test, *P*-value = 0.468 (age, notification centre, citizenship and HIV status); Classification table 84% correctly classified; area under receiver operating characteristic (ROC) was 84.5

## References

[1] World Health Organization (WHO) (2021). Global tuberculosis report.

[2] Roberts CA, Davies PD, Blevins KE, Stone AC, Plomp KA (2022). Chapter 10. Preventable and curable, but still a global problem: tuberculosis from an evolutionary perspective. Palaeopathology and evolutionary medicine: an integrated approach.

[3] Visca D, Ong C, Tiberi S, Centis R, D’ambrosio L, Chen B (2021). Tuberculosis and COVID-19 interaction: a review of biological, clinical and public health effects. Pulmonology.

[4] Avoi R, Liaw YC (2021). Tuberculosis death epidemiology and its associated risk factors in Sabah, Malaysia. Int J Environ Res Public Health.

[5] Awang H, Ning GS, Ahmad MH, Mohamed KA, Zuber MFM, Embong K (2022). Epidemiology of mortality among tuberculosis patients on treatment in Terengganu state of Malaysia. J Health Trans Med.

[6] Department of Statistics Malaysia (2020). Manjung: district of Malaysia in Perak report.

[7] Government of Malaysia (1988). Undang-undang Malaysia: Akta Pencegahan dan Kawalan Penyakit Berjangkit 1988 (Act 342).

[8] Ministry of Health (MoH) Malaysia (2021). Management of tuberculosis.

[9] Saunders MJ, Tovar MA, Collier D, Baldwin MR, Montoya R, Valencia TR (2019). Active and passive case-finding in tuberculosis-affected households in Peru: a 10-year prospective cohort study. Lancet Infect Dis.

[10] Gatechompol S, Kawkitinarong K, Suwanpimolkul G, Kateruttanakul P, Manosuthi W, Sophonphan J (2019). Treatment outcomes and factors associated with mortality among individuals with both TB and HIV in the anti-retroviral era in Thailand. J Virus Erad.

[11] Ahmad N, Baharom M, Aizuddin AN, Ramli R (2021). Sex-related differences in smear-positive pulmonary tuberculosis patients in Kuala Lumpur, Malaysia: prevalence and associated factors. PloS One.

[12] Tok PSK, Liew SM, Wong LP, Razali A, Loganathan T, Chinna K (2020). Determinants of unsuccessful treatment outcomes and mortality among tuberculosis patients in Malaysia: a registry-based cohort study. PloS One.

[13] Chakaya J, Khan M, Ntoumi F, Aklillu E, Fatima R, Mwaba P (2021). Global tuberculosis report 2020—reflections on the global TB burden, treatment and prevention efforts. Int J Infect Dis.

[14] Hannah HA, Miramontes R, Gandhi NR (2017). Sociodemographic and clinical risk factors associated with tuberculosis mortality in the United States, 2009–2013. Public Health Rep.

[15] Beaumont A, Doumbia A, Château N, Meynard J, Pacanowski J, Valin N (2022). Why are people still dying of drug-susceptible TB in Paris in the 21st century?. Int J Tuberc Lung Dis.

[16] Sharma M, Onozaki I, Nunn P (2021). TB in older people in Asia: why it is important. Int J Tuberc Lung Dis.

[17] Pacelli B, Zengarini N, Broccoli S, Caranci N, Spadea T, Di Girolamo C (2016). Differences in mortality by immigrant status in Italy. Results of the Italian network of longitudinal metropolitan studies. Eur J Epidemiol.

[18] Giorgi Rossi P, Mantovani J, Ferroni E, Forcina A, Stanghellini E, Curtale F (2009). Incidence of bacterial meningitis (2001–2005) in Lazio, Italy: the results of a integrated surveillance system. BMC Infect Dis.

[19] Norredam M, Olsbjerg M, Petersen J, Bygbjerg I, Krasnik A (2012). Mortality from infectious diseases among refugees and immigrants compared to native Danes: a historical prospective cohort study. Trop Med Int Health.

[20] Musaazi J, Sekaggya-Wiltshire C, Kiragga A, Kalule I, Reynolds S, Manabe Y (2019). Sustained positive impact on tuberculosis treatment outcomes of TB-HIV integrated care in Uganda. Int J Tuberc Lung Dis.

[21] Marks S, Magee E, Robison V (2011). Patients diagnosed with tuberculosis at death or who died during therapy: association with the human immunodeficiency virus. Int J Tuberc Lung Dis.

[22] Straetemans M, Bierrenbach AL, Nagelkerke N, Glaziou P, van der Werf MJ (2010). The effect of tuberculosis on mortality in HIV positive people: a meta-analysis. PLoS One.

[23] Mendelson M (2007). Diagnosing tuberculosis in HIV-infected patients: challenges and future prospects. Br Med Bull.

